# Paramagnetic Intrinsic Defects in Polycrystalline Large-Area 2D MoS_2_ Films Grown on SiO_2_ by Mo Sulfurization

**DOI:** 10.1186/s11671-017-2008-x

**Published:** 2017-04-20

**Authors:** A. Stesmans, S. Iacovo, D. Chiappe, I. Radu, C. Huyghebaert, S. De Gendt, V. V. Afanas’ev

**Affiliations:** 1 Department of Physics and Astronomy, University of Leuven, 3001 Leuven, Belgium; 2 Imec, Kapeldreef 75, 3001 Leuven, Belgium

**Keywords:** Large-area 2D molybdenum disulfide, Synthetic 2D molybdenum disulfide, Point defects, Intrinsic defects, Grain boundaries, Electron spin resonance

## Abstract

A low-temperature electron spin resonance study has been carried out on large-area high-purity polycrystalline two-dimensional few monolayer (ML) 2H MoS_2_ films synthesized by sulfurization of Mo layers, with intent to atomically assess mobility-degrading detrimental point defects. This reveals the presence of a distinct previously unreported anisotropic defect of axial symmetry about the *c*-axis characterized by *g*_//_ = 2.00145 and *g*_⊥_ = 2.0027, with corresponding density (spin S = ½) ~3 × 10^12^ cm^−2^ for a 4 ML thick film. Inverse correlation of the defect density with grain size points to a domain boundary associated defect, inherently incorporated during sample growth. Based on the analysis of ESR signal features in combination with literature data, the signal is tentatively ascribed to the a (di)sulfur antisite defect (S or S_2_ substituting for a Mo atom). Beset by these defects, the grain boundaries thus emerge as an intolerable threat for the carrier mobility and layer functionality.

## Background

Next to graphene, MoS_2_ is touted as a post-Si semiconductor that may revolutionize electronics because of distinct advantages [1–3]. Unlike graphene, a two-dimensional (2D) MoS_2_ monolayer (ML) is a 1.85-eV direct bandgap semiconductor [4], which makes it most appropriate for integrated logic and optoelectronic applications. The practical realization of any of these will require synthesis of large-area high-quality 2D MoS_2_ layers, stimulating research for optimal production techniques [5, 6].Yet, related with the growth process, inevitably present structural defects, such as point defects and grain boundaries, manifestly degrade the performance, including electrical mobility degradation to values still one or two orders of magnitude below the theoretical one of ~410 cm^2^/Vs for electrons [1, 5, 7–9]. Many kinds of intrinsic point defects have been theoretically [5, 10–15] and experimentally [5, 11, 16] assessed including vacancies, antisites, and interstitials, with the sulfur vacancy, V_S_—an acceptor [10, 17]—emerging with the lowest formation energy [5, 10, 11]. Using high-resolution scanning transmission electron microscopy (STEM) techniques, several defects have been experimentally “visualized” [5, 11, 16] and their occurrence statistically analyzed [5].

Obviously, adequate steering of the realization of high-quality 2D crystalline films requires, besides identification, also quantification of the various type defects before these can put under control. In an exploring approach, the current work deals with high-purity large-area 2D MoS_2_ polycrystalline layers synthesized by sulfurization of Mo films, focusing on occurring detrimental point defects with the intent to atomically assess and quantify these by means of electron spin resonance (ESR), an exclusive tool for that purpose. A pristine signal, of substantial intensity, is revealed, suggested to originate from a native defect related with grain boundaries, thus unveiling a severe threat to performance.

### Methods

**Fig. 1:**
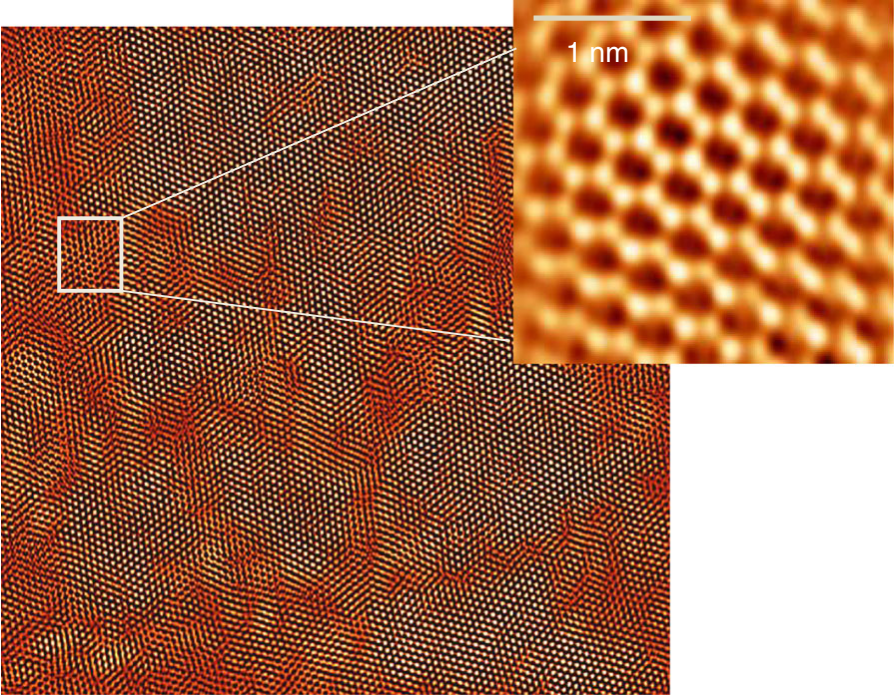
Plane-view STEM image of a large-area MoS_2_ film synthesized on SiO_2_ by sulfurization of a predeposited (sputtered) Mo layer at 800 °C in H_2_S (100 mbar; 30 min) exposing a uniform polycrystalline film of average grain size ~20 nm; The zoomed in picture shows the hexagonal structure and trigonal prismatic atomic arrangement geometry

The starting sample substrates were 2 cm × 2 cm two-side polished Cz-(100)Si slices (B-doped; ~1 Ω cm; ~100 μm thick) thermally oxidized (~50 nm thick) at both sides, and subsequently subjected to heating in H_2_ (1 atm; 6 N pure; 430 °C) to ESR-inactivate the inevitable interfacial Si dangling bond (DB) defects (P_b_-type centers) [18]. On these substrates, a thin Mo layer with thickness in the range 0.2–0.5 nm, as measured by quartz monitored weighing, was sputtered at a deposition rate of 0.01 nm/s from a high-purity Mo source in high vacuum, followed by MoS_2_ layer synthesis through sulfurization at 800 °C for 30 min in pure H_2_S at a pressure p_H2S_ of 100 mbar (process a) or 10 mbar (process b), according to the chemical reaction Mo + 2H_2_S → MoS_2_ + 2H_2_ (*g*). As exposed by STEM analysis, this resulted in continuous large-area 2D MoS_2_ polycrystalline films, with ~20–40 nm sized grains, in the 2H phase (hexagonal symmetry, two MoS_2_ layers per repeat unit, and Mo in trigonal prismatic coordination; D_3h_ point group). This is illustrated in Fig. 1, showing a plane-view STEM image of a large area MoS_2_ film obtained by sulfurization process a, the layer being characterized by an average grain size of ~20 nm across. Cross-sectional TEM observations show that film thicknesses of up to 4 MLs, rather uniform, are obtained with the molecular planes preferentially aligned parallel to the SiO_2_ substrate surface. More details about the synthesis, morphological and structural analysis, and performance can be found elsewhere [19].

Three samples were examined: a first with one MoS_2_ monolayer (1MLa) grown at p_H2S_ = 100 mbar, a second 4-layer thick one (4MLa) grown at *p*_H2S_ = 100 mbar, and a third 4-layer one (4MLb) grown at p_H2S_ = 10 mbar. Atomic force imaging of the 4ML samples shows an increase in average grain size from ~20 to ~40 nm by reducing p_H2S_. From measurements on bottom-gated transistors fabricated using these large-area 4ML MoS_2_ films, extrinsic low-field field-effect carrier mobilities of ~0.001 and ~0.02 cm^2^/Vs were obtained, respectively.

For ESR purposes, the as-received samples were mechanically cleaved into 2 mm × 10 mm slices, with their 10-mm edge along the Si [0‾11] direction. In assembling an ESR sample (typically ~20 slices), full wafer coherence in crystallinity was maintained through stacking slices with their Si substrate [0‾11] direction all “up” in the bundle. Defects were characterized using conventional low-temperature X, K, and Q-band ESR spectroscopy [18]. Some samples were additionally subjected to thermal treatment in vacuum (*p* ≤ 5 × 10^−6^ mbar) at *T* = 330 °C for appropriate times.

## Results

**Fig. 2:**
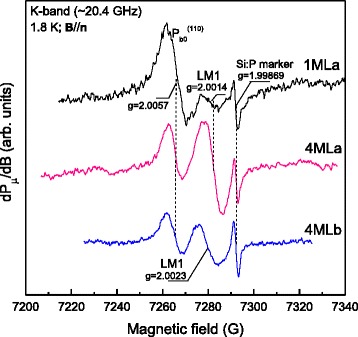
K-band ESR spectra observed at 1.8 K using *P*_μ_ ~ 25 nW for **B**//**n** on three 2D MoS_2_/SiO_2_/(100)Si entities, showing the observation of the LM1 signal (at *g* ~ 2.0014 in the process-a samples). The signal at *g* = 1.99875 stems from a co-mounted Si:P marker sample, also used for field axis alignment of the spectra. The spectra have been normalized to equal Si:P marker intensity and sample area

**Fig. 3:**
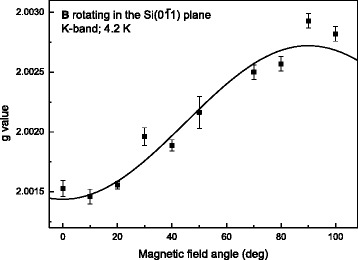
*g* map of the LM1 defect in MoS_2_ sample 4MLa inferred from K-band ESR measurements at 4.2 K for **B** (at angle φ_B_ with **n**) rotating in the Si substrate (0‾11) plane. The solid curve represents an optimized fitting of expression *g*(φ_B_) = [*g*_//_^2^cos^2^(φ_B_) + *g*_⊥_^2^sin^2^(φ_B_)]^½^ for a center of axial (C_3v_) symmetry giving *g*_//_ = 2.00145 and *g*_⊥_ = 2.0027

Figure 2 presents an overview of representative first-derivative (dP_μ_/dB, where *P*_μ_ is the applied microwave power and **B** the magnetic field) K-band ESR spectra measured for **B** parallel to the [100] surface normal (**n**) at 1.8 K in the *g* = 2.018–1.98 range (~130 G scan) for the three types of samples studied. Two main signals are observed: The first, observed at *g* ~ 2.0057 with peak-to-peak width ΔB_pp_ ~ 8G for **B**//**n** and exhibiting distinct *g* factor anisotropy (not shown), stems from the known anisotropic Si DB P_b0_^(110)^ defects at the (110)Si/SiO_2_ interface, well expected from the (011) and (0‾11) cleavage edges of the (100)Si slices. The signal is identically observed, and the only one, on a SiO_2_/(100)Si/SiO_2_ reference sample without MoS_2_ layers on top. Of key interest is the 2nd signal of ΔB_pp_ ~ 7G, labeled LM1, appearing at *g* ~ 2.0014(2) in the process-a (1MLa, 4MLa) samples. Field angular measurements for **B** rotating in the Si (0‾11) plane (**B** at angle φ_B_ with **n**) result in the *g* map shown in Fig. 3, revealing anisotropy. The map points to a defect of axial (C_3v_) symmetry where the axial (*g*_//_) axis is restricted to only one direction with respect to the sample morphology, i.e., parallel to the MoS_2_ layer normal **n**, with potentially allowed other crystallographically equivalent defect orientations in a bulk crystal not occurring. Optimized fitting to axial symmetry yields *g*_//_ = 2.00145 and *g*_┴_ = 2.0027.

The defect appears in substantial densities, with inferred values of ~ 3 × 10^11^ and 3 × 10^12^ cm^−2^ for the 1MLa and 4MLa samples, respectively. A similar signal, with corresponding density ~0.8 × 10^12^ cm^−2^, is observed in the low *p*_H2S_ = 10 mbar sample 4MLb at *g* ~ 2.0023 but now behaving more isotropic. Here, we may notice that the latter value coincides with the *g*-matrix trace *g* = (*g*_//_ + 2 *g*_┴_)/3 = 2.00228. Within experimental accuracy, the signal remains unchanged after vacuum treatment of the sample at 330 °C for ~150 min.

**Fig. 4:**
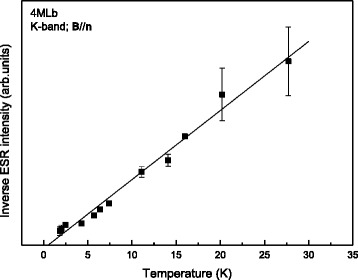
Plot of the inverse ESR intensity of the LM1 signal from sample 4MLb vs temperature. The data point to a spin system behaving closely paramagnetic (*χ* ^−1^ ∝ *T*), characterized by a Curie temperature *T*_c_ = 0.5 ± 0.5 K as obtained from the optimized linear fitting (*solid line*) of the Curie-Weiss law *χ* ^-1^ ∝ (*T* − *T*_c_)

As illustrated by the plot in Fig. 4 of the inverse signal intensity I (area under the absorption curve ∝ magnetic susceptibility *χ*) vs. *T*, the signal behaves almost perfectly paramagnetic [*χ* ∝ *T* ^−1^] within experimental uncertainty; Fitting of the Curie-Weiss law *χ* ∝ (*T* − *T* ^−1^), gives a Curie temperature *T*_C_ = 0.5 ± 0.5 K, i.e., close to 0, which points to a spin system of localized defects with negligible mutual interaction. It is indicative of a defect system distributed in a dilute manner with negligible defect clustering. As the LM1 signal is not observed in the thermal SiO_2_/(100)Si/SiO_2_ control sample, it should originate from the 2D MoS_2_ layer, and based on the high-purity level attained with synthesized MoS_2_ layers, it is reasonable to ascribed it to a native intrinsic defect.

## Discussion

Having revealed a first exclusive paramagnetic point defect pertaining to 2D MoS_2_ layers, it remains to address its atomic nature. Dealing with 2D layers deposited on SiO_2_/Si substrates, in search of its atomic character, there is the basic question about the location of the defect at/in the MoS_2_ layers studied, for which we face several possibilities, i.e., edge defects, ad-atom (surface) centers, or “inner” layer defects. Here, as a first inference, the kind of rather high density of defects observed precludes right away, for a continuous layer, these to originate from the MoS_2_ layer edges. Next, the fact that the LM1 signal is observed not to be affected by treatment in vacuum at 330 °C (~150 min), would exclude the observed signal neither to concern an ad-atom center [10]. As to its location, this thus leads us to considering a more inner layer positioned *intrinsic* defect, in which case the abundant grain boundaries emerge as primary suspect. Some independent support for this hypothesis comes from the fact that the signal is not observed by independent high-sensitivity ESR measurements on a natural as-received 2H MoS_2_ crystal (not shown). The suggestion is further supported by the observed decrease in defect density for the 4MLb MoS_2_ layer compared to the 4LMa one, i.e., in correlation with the increase in average grain size (~20 vs. ~40 nm), with attendant substantial reduction in amount of grain boundaries—and hence in the total of associated point defects—and drastic improvement in carrier mobility [19].

Reliable atomic identification generally would require the observation of ESR hyperfine (hf) structure, which in the current case should come from the isotopes ^33^S (nuclear spin *I* = 3/2; 0.75% abundance) and ^95^Mo and ^97^Mo (both *I* = 5/2; 25.5% added abundance). Yet, despite spectroscopic realization of a signal-to-noise ratio >300 for the LM1 Zeeman signal, no clear hf structure could be resolved. Based on the involved Mo nuclear magnetic moment strength μ_n_ = g_n_β_n_I = −0.37β_n_I (where *g*_n_ and β_n_ represent the nuclear *g* factor an nuclear magneton, respectively) [20] and natural isotopic abundance, this makes it unlikely the defect to concern a Mo-centered unpaired electron defect.

No previous report of an LM1-like ESR spectrum in MoS_2_ could be traced in the literature. Also, based on *g* value considerations, literature search would indicate the signal not to originate from unsaturated sulfur atoms (radicals) or Mo atoms in the formal 5+ oxidation state [21].

Obviously, regarding intrinsic defects in the two-atom MoS_2_ material, there are various possible variants. Using the annular dark-field (ADF) STEM technique, several works have recently managed atomic-scale visualization of intrinsic structural defects—point defects, dislocations, grain boundaries, edges—in MoS_2_ monolayer prepared by various methods, including mechanical exfoliation [5] from natural MoS_2_ samples, chemical vapor deposition [5, 11], and physical vapor deposition (PVD) [5]. There, besides the various types of vacancy centers (i.e., V_S_, V_Mo_, V_S2_, V_MoS3_, V_MoS6_), eye catching was the demonstration of the presence of various antisite centers like S_Mo_ (a sulfur atom substituting for a Mo site), S2_Mo_, Mo_S_, and Mo_S2_. ADF-STEM observations showed that the Mo_S2_ antisite defects, followed by Mo_S_, are far dominant defects in PVD MoS_2_ monolayers reaching densities of ~2.8 × 10^13^ cm^−2^. Substantial densities of S_Mo_ were found in mechanically exfoliated MoS_2_ monolayers. Thus, antisites have been atomically resolved in various instances [5, 11], also in connection with grain boundaries.

It has been concluded that antisites should play an important role in the dislocation and grain boundary structures [10, 14, 22]. Also, antisites are reported to have a strong effect on the phonon-limited mobility of electrons, far more drastic than V_S_ or V_S2_. [5]. With these findings as background, in light of the distinct polycrystalline nature of the MoS_2_ films currently studied, it is suggested the LM1 signal to originate from antisite defects associated with grain boundaries, where based on the salient ESR properties, the S_Mo_ or S2_Mo_ antisites come to the fore as most likely.

If correct, then based on the observed defect density and measured average grain size, we would have ~1.5–3.5 antisite defects per 10-nm length of grain boundary. Obviously, these will drastically devastate the carrier mobility. Accordingly, this mandates that the production method is to be upgraded so as to drastically reduce their generation, or else, if unavoidably incorporated, an appropriate method should be developed that enables efficient and robust electrical inactivation (passivation) of these defects.

It should be noticed that, given the large formation energy (E_form_) involved [14], the grain boundaries, and likely the associated antisite defects as well, come as “non-equilibrium” structures introduced by sample growth modalities and history. Their appearance is seen as inherent to the specific synthesis method, carried out at relatively high *T* (~800 °C).

Among the antisites theoretically assessed, i.e., Mo_S_, S_Mo_, S2_Mo_, Mo_S2_, E_form_ of the former two is anticipated to be smallest [5, 10, 11]. Yet, when dealing with MoS_2_ grown under S-rich conditions, the E_form_ (~4 eV) of S_Mo_ emerges as resolutely the lowest [10]. The formation energy is further calculated to be drastically lowered at grain boundaries compared to the grain interior [23]. Accordingly, given the S-rich growth condition of the currently studied the MoS_2_ layers, this would favor assignment of the ML1 signal to S_Mo_ defects.

This leads to the proposition that in the studied polycrystalline synthetic 2D MoS_2_ layers, the grain boundary dislocation are the source of a large density of paramagnetic defects, that on comparative grounds, will unacceptably impair the layer’s electrical and optoelectronic functionality.

Finally, when overviewing the results of the studied 4ML MoS_2_ samples, we notice that the observed strong reduction (~4 times) with increasing grain size is in line with the measured drastic improvement in carrier mobility; It would indicate, not unexpectedly, that the grain boundaries with embedded (antisite) point defects are at the basis of the strong deterioration of the carrier mobility.

## Conclusions

In conclusion, low-T ESR study has revealed that the grain boundaries in continuous large-area few-layer 2H MoS_2_ films synthesized by sulfurization of Mo films have incorporated an excessive density of paramagnetic defects, a fact, as it appears, inherent to the preparation method applied. Occurring in densities of up to ~3 × 10^12^ cm^−2^ for 4 ML-thick MoS_2_ films, on comparative grounds, these defects will inaptly impair and limit the charge carrier mobility and limit optoelectronic functionality. Led by stepwise elimination, the originating center is tentatively assigned to the S_Mo_ or S2_Mo_ antisite, “inherently” associated with grain boundaries, an intrinsic structural defect of which the incorporation emerges as inherent to the growth method used. With respect to the fabrication method applied, the incorporation of such type of defect in distinct quantities may perhaps not come as a surprise. The advanced hypothesis is to be subjected to verification by first-principle theoretical simulations able to reliably calculate *g* values of point defects in MoS_2_ to sufficient accuracy.

Obviously, defects in 2D MoS_2_ layers are generally detrimental for, and may cause undue large variations in, electrical and optical properties, and should thus be maximally suppressed or electrically inactivated, the more so for antisite-type defects. The currently gained information is expected to be of use on the road to refine and optimize the layer synthesis procedure, with the view to come to large area, continuous 2D transition metal dichalcogenide (TMD) layers of uniform device-grade quality throughout. As layered TDMs have very similar structures, the structural defect revealed in this work may be expected to surface in other 2D TDMs as well, e.g., WS_2_, particularly when manufactured in a similar way.

## Abbreviations

2D: Two dimensional
Cz: Czochralski
DB: Dangling bond
ESR: Electron spin resonance
ML: Monolayer
ADF: Annular Dark Field
STEM: Scanning transmission electron spectroscopy
TMD: Transition metal dichalcogenides
